# *Drosophila* Cyclin G Is a Regulator of the Notch Signalling Pathway during Wing Development

**DOI:** 10.1371/journal.pone.0151477

**Published:** 2016-03-10

**Authors:** Anja C. Nagel, Jutta Szawinski, Mirjam Zimmermann, Anette Preiss

**Affiliations:** Institut für Genetik, Universität Hohenheim, Garbenstr. 30, 70599 Stuttgart, Germany; National Institutes of Health (NIH), UNITED STATES

## Abstract

Notch signalling regulates a multitude of differentiation processes during *Drosophila* development. For example, Notch activity is required for proper wing vein differentiation which is hampered in mutants of either the receptor Notch, the ligand Delta or the antagonist Hairless. Moreover, the Notch pathway is involved in several aspects of *Drosophila* oogenesis as well. We have identified *Drosophila* Cyclin G (CycG) as a molecular interaction partner of Hairless, the major antagonist in the Notch signalling pathway, *in vitro* and *in vivo*. Loss of CycG was shown before to cause female sterility and to disturb the architecture of the egg shell. Nevertheless, Notch dependent processes during oogenesis appeared largely unaffected in *cycG* mutant egg chambers. Loss of CycG modified the dominant wing phenotypes of *Notch*, *Delta* and *Hairless* mutants. Whereas the *Notch* loss of function phenotype was ameliorated by a loss of CycG, the phenotypes of either *Notch* gain of function or of *Delta* or *Hairless* loss of function were enhanced. In contrast, loss of CycG had only a minor effect on the wing vein phenotype of mutants affecting the EGFR signalling pathway emphasizing the specificity of the interaction of CycG and Notch pathway members.

## Introduction

The Notch signalling pathway is highly conserved in higher animals and humans and is required for the cellular differentiation in a multitude of tissues. Most importantly, Notch signalling drives the process of lateral inhibition, where a primary cell is singled out from a group of equipotential cells to obtain a different cell fate (reviewed in [1–3]). In *Drosophila*, the Notch receptor is activated by the membrane bound ligands Delta (Dl) and Serrate (Ser), respectively. By expressing the Notch ligand, the primary cell activates the Notch receptor pathway in the neighbouring cells, thereby forcing them into an alternative cell fate. Classical examples for this process are the selection of neuronal precursors in the central and peripheral nervous system or the establishment of wing veins that are slimmed to their final width from a broader field of provein cells [1–5]. Moreover, Notch signalling is activated in specific cells that subsequently change their fate, which has been dubbed ‘inductive signalling’ (reviewed in [1–3]). A typical example is the induction of the dorso-ventral boundary in the wing primordium eventually giving rise to the wing margin. Hereby, Notch is precisely activated along the dorso-ventral boundary by Delta and Serrate that are unevenly expressed in the ventral and dorsal primordial compartments, respectively [6–7]. Furthermore during oogenesis, the somatic polar follicle cells and the stalk follicle cells are specified by an inductive Delta signal derived from the germ line [8–9].

We are interested in the role of Cyclin G (CycG) in *Drosophila*. CycG belongs to the atypical cyclins that function in a variety of processes including transcription, cellular differentiation or stress response (reviewed in [10]). There are two *cycG* genes in mammals (CCNG1/2) with apparently disparate roles in the regulation of cell proliferation, growth and stress resistance [11–17]. CycG1 was originally identified as one of the first p53 targets, and it interacts with several cell cycle regulators including MDM2, ARF and RB [12,18,19]. Moreover, CycG1 binds to cdk5 and GAK *in vitro* and *in vivo* [20]. Most interestingly, both CycG1 and CycG2 physically interact with the B’-subunit of protein phosphatase 2A (PP2A), and it has been proposed that CycG1 acts as a specificity factor of PP2A, thereby inhibiting p53 activity [15,21]. There is a single *cycG* homologue in *Drosophila*, and the encoded CycG protein is both cytoplasmic and nuclear [22]. CycG was shown to likewise interact with the PP2A B’-subunit Widerborst *in vitro* and *in vivo* as well as genetically [23–25]. In addition, binding to the Cyclin dependent kinases Cdk2, Cdk4 and Cdk5 was observed *in vitro* and in cell culture, presumably explaining the effects of a manipulation of CycG expression levels on cell cycle length in the fly [24,26].

Recently, we generated a null mutation in the *cycG* gene by homologous recombination. We found that CycG is not essential for fly viability but for female fertility, since mutant females are sterile and produce ventralized eggs [27]. The egg phenotype of *cycG* mutants results from a downregulation of EGFR signalling due to defects in the translation of *grk* mRNA in the oocyte [27, 28]. In fact CycG is involved in the meiotic checkpoint control: in the absence of CycG, the meiotic checkpoint is activated, indirectly affecting *grk* translation and resulting in ventralized eggs [27]. In ovaries, CycG protein is associated with BRCA2 and Rad9, both well established components of the 9-1-1 complex, which engages in DNA double strand break sensing and checkpoint activation (reviewed in [29]). The interaction of CycG with the 9-1-1 complex suggests a role for CycG in early processes of DNA double strand repair [27]. In addition to its role during meiosis, we found CycG to be required for imaginal development as well, where it acts as a positive regulator of InR/TOR-signalling at the level of Akt1 kinase. Accordingly, *cycG* homozygous mutants phenocopy *TOR* mutants in several aspects, notably in growth retardation, defective lipid metabolism and energy homeostasis [25].

Here we show that CycG is a molecular interaction partner of Hairless *in vitro* and *in vivo*. Hairless is the major antagonist of the Notch signalling pathway in *Drosophila* and acts by transcriptional repression of Notch target genes (reviewed in [30]). Hence, CycG may play additional roles during *Drosophila* somatic development. Indeed the molecular interaction data were confirmed by genetic interactions during wing development, where both Notch mediated processes of wing vein specification and wing margin formation are affected. In addition, minor modifications of EGFR-related wing vein defects in the *cycG* mutants were observed. In contrast, we have no indication that the activity of CycG influences Delta–Notch signalling during early oogenesis. Here we addressed the specification of polar follicle cells and stalk cells that both appear normal in the *cycG* mutant. However, we find alterations in the process of dorsal appendage formation, where Notch expression is downregulated in *cycG* mutant egg chambers. This downregulation is presumably a consequence of the impaired EGFR signalling activity in the *cycG* mutant follicle, resulting in a mis-specification of the dorsal roof cells, rather than a direct requirement of CycG for Notch signalling activity. Altogether, we provide evidence for a molecular and genetic interaction of CycG with the Notch signalling pathway in somatic tissues during wing development of the fly.

## Materials and Methods

### *In vitro* protein-protein interactions

As described earlier [31], pEG-HFL encompassing the complete H cDNA [32], was used as bait in a yeast two-hybrid screen on the *Drosophila melanogaster* embryonic cDNA library RFLYI [33]. With 30% of the total, clone pJG6-9 was the second most frequent clone isolated in this screen; pJG6-9 encoded a full length *cycG* cDNA corresponding to transcript RA (www.flybase.org). It contained an 80 nucleotide leader sequence but retained an open reading frame and expressed a fusion protein of the expected size extended by 26 amino acids. The *cycG* cDNA sequence was PCR-amplified using the primer pair 5’-CAA AAC GAA TTC ATG CAG ATA CTG ATC AAA ACG CA-3’ and 5’-TAA TGC TGC TTG CTG TCG CCT CGA GCT AAC ATT-3’ and cloned as *Eco* RI and *Xho* I restriction fragment into likewise linearized pEG202 and pJG vectors [34]. Clones were sequence verified, and protein expression in yeast cells was tested in Western blots with antibodies directed against HA (clone HA-7, Sigma-Aldrich) and LexA (61–001, Bio Academia). The pEG and pJG CycG constructs start at position -17 compared to the published sequence (www.flybase.org). In subsequent qualitative and quantitative yeast two-hybrid assays, they were indistinguishable from pJG6-9. To allow for the reverse experiments, a full length H cDNA was PCR-amplified using the primer pair 5’-CAA GGT ACC AAA TGG CCC TGC TTA ATG A-3’ and 5’-GTG GGT ACC TCA TGT CTT TGA CAG ATT C-3’ and cloned as *Kpn* I fragment into likewise linearized VP16-vector [34]. Yeast two-hybrid protein interaction assays were performed as previously described using full length Hairless pEG HFL, and the Hairless deletion constructs pEG H-C1, H-C2, H-C3, H-CX and H-C6 [32]. Quantitative beta-galactosidase activity assays were done according to standard protocols as described before [32, 35]: Miller units were calculated from the ratio of substrate turnover to cell density (1000x OD_420_/time [min] x volume [ml] x OD_600_). The CycG deletion constructs cloned in pJG-vector 1–215 and 215–566 have been described before [22].

### *In vivo* protein-protein interactions

Co-immunoprecipitations were performed as described before [36] using protein extracts of approximately 500 wild type embryos. Immunoprecipitation were done with polyclonal rabbit anti-Hairless A or guinea pig anti-CycG antisera in a 1:250 dilution [22, 37]. For detection of precipitates on Western blots, the respective antisera were from rat and used at 1:500 dilution [22, 37]. Secondary antibodies coupled to alkaline phosphatase were diluted 1:200 (Jackson Laboratories, Dianova).

### Genetic interaction studies

The *cycG*^*CreD*^ allele was derived from the *cycG*^*HR7*^ null allele [27]: the proximal *cycG* gene copy and the *white*^+^ marker gene were excised with help of I-*CreI* exactly as described before [38]. The *cycG*^*CreD*^ mutant allele was molecularly verified by Southern blotting and PCR analysis: it contains the distal mutant *cycG* gene copy. No CycG protein was detected on Western blots, i.e. *cycG*^*CreD*^ behaved like *cycG*^*HR7*^ [27, 28].

Flies were raised on standard cornmeal food at 18°C; crosses were kept at 25°C. Standard genetic procedures were used to generate double mutant combinations. Recombinants were assayed by PCR for the presence of the *cycG* mutant allele using the primer pair cycG10440UP (5’- AGG CCA GCC CTC ACA ATG TCT GTC-3’) and cycG12330LO (5’-TTG GAC CAA AGA ACT TTG CGG CAG-3’). The following mutant alleles were used: *cycG*^*HR7*^ [27], *cycG*^*CreD*^, *cycG*^*eoC*^ [25], *Dl*^*B2*^ [39], *H*^*P8*^ [37], Df(1)-N5419 [40], *N*^*cos479*^ [41], *Su(H)*^*∆47*^ [42], *net*^*1*^, *ve*^*1*^ and *vn*^*1*^ [43]. Polar cells were visualized using the *neur*^*A101*^-lacZ reporter line [8]. Oregon-1, Berlin-K, *w*^*1118*^ and the balanced siblings served as controls. Further information on fly stocks can be obtained from flybase.org.

### Histology and histochemistry

Wings were dehydrated in ethanol and mounted in Euparal (Roth) for microscopic examination with a Zeiss Axioskop (Carl Zeiss) as described earlier [44]. Pictures were taken with a Pixera digital camera (Optronics), using the Pixera Viewfinder Version 2.0 software. The width of veins was measured using Image J programme. Statistical significance of probes was determined using Student's T-test (http://www.physics.csbsju.edu/stats/t-test.html) with p-values: p>0.05 (not significant, n.s.); p<0.05 (weakly significant; *); p<0.01 (significant; **); p<0.001 (highly significant; ***).

Antibody staining on wing imaginal discs and ovaries was done as outlined earlier [27, 45]. The following monoclonal antisera were obtained from Developmental Studies Hybridoma Bank: Mouse anti-beta-Galactosidase (1:200; JIE7, developed by T.L. Mason and J.A. Partaledis), mouse anti-Delta (1:50; C594.9b) and mouse anti-Notch intracellular domain (1:50; C17.9C6) (both developed by S. Artavanis-Tsakonas) and anti-Broad core (1:50; 25E9.D7, developed by G. Guild). Goat polyclonal secondary antibodies (1:200) coupled to either Fluorescein isothiocyanate (FITC), Cyanine 3 (Cy3) or Cyanine 5 (Cy5) were purchased from Jackson Laboratory (Dianova). Fluorescently labelled tissue was mounted in Vectashield (Vector Lab). Images were collected with a Zeiss Axiophot linked to a Bio-Rad MRC1024 confocal microscope using LaserSharp2000TM software (Carl Zeiss). Pictures were processed and assembled using Corel Photopaint and Corel Draw software.

## Results

### Identification of CycG as an interaction partner of Hairless

**Fig 1:**
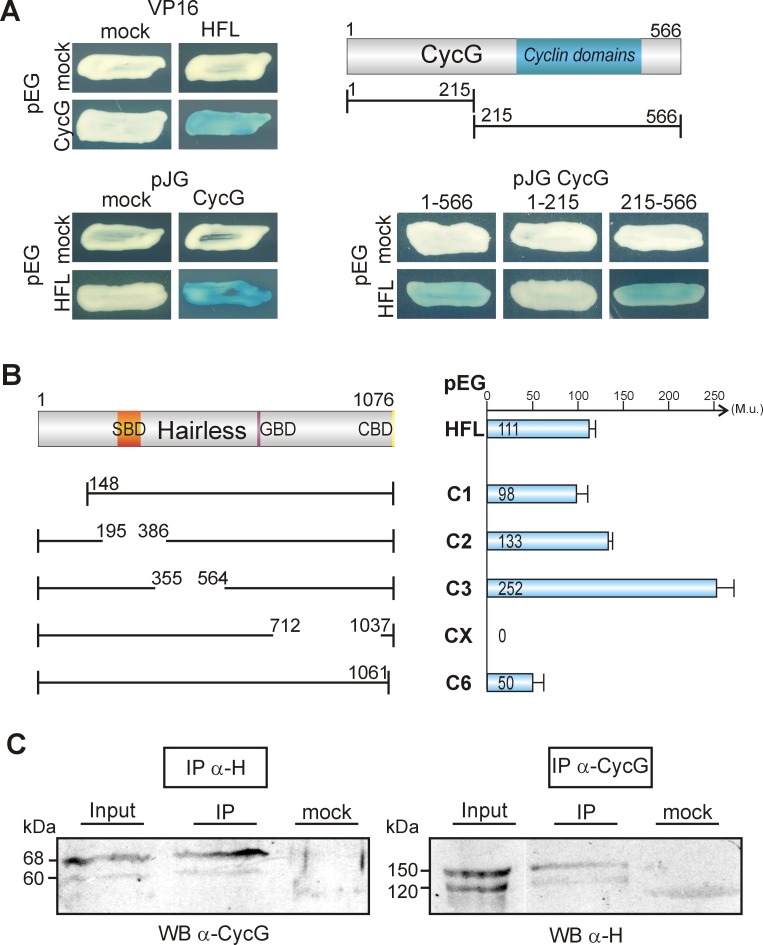
CycG protein interacts with Hairless protein *in vitro* and *in vivo*. **(A)** Yeast two-hybrid assay for pairwise interactions between CycG and Hairless (HFL): interaction is observed irrespective of the orientation of the assay (blue coloured colonies). pEG vector expressed the DNA-binding protein fusion; pJG and VP16 vectors the DNA-activation domain fusions. Empty vectors served as negative control (mock). Of the two CycG deletion constructs 1–215 and 215–566, only the latter containing the Cyclin domains (blue) binds to HFL. **(B)** Left panel shows the constructs: a sketch of the full length Hairless protein with the Su(H) binding domain (SBD, orange), the Gro binding domain (GBD, purple) and the CtBP binding domain (CBD, yellow). Numbers correspond to the codons contained in the construct according to 35, 37]. Right panel: Quantification of the interaction between CycG, full length Hairless (HFL) and Hairless deletion constructs as indicated. Values represent Miller units (M.u.), determined as outlined in the Methods section. Note that the C-terminal deletion H-CX shows no interaction, whereas H-C6 shows a strongly reduced binding to CycG. **(C)** Co-immunoprecipitations (IP) were performed on embryonic extracts using polyclonal antisera as indicated, directed either against Hairless or CycG. Co-precipitates were detected by Western blot (WB) in both experiments indicating *in vivo* interaction of the two proteins. Mock control contained no primary antiserum.

To further our understanding of Hairless functions, a yeast two-hybrid screen for potential interaction partners of Hairless was performed. As bait we used the full length Hairless cDNA (HFL) to screen a library containing cDNA from 0–6 hours old embryos [31, 33]. This screen yielded a total of 57 clones, 17 of which turned out to contain a full length sequence of CycG. To confirm the specificity of the interaction, both CycG and H cDNA were each cloned into pEG, pJG or VP16 yeast vector, and the interaction tests were repeated in both orientations (Fig 1A). Hairless binding was mapped to the C-terminal half of CycG which contains the Cyclin domains [22]. In Hairless, the CycG interaction domain was mapped to the C-terminal H-CX domain (Fig 1B), since the corresponding deletion completely failed to bind to CycG. In contrast, neither deletion H-C1, H-C2, H-C3 nor H-C6 destroyed the binding to CycG, but H-C6 bound markedly weaker (Fig 1B). Until now no precise role has been assigned to the H-CX domain: it contains several boxes of high sequence conservation [46, 47] and interacts with Runt, a regulator of embryonic segmentation [48] as well as with Pros26.4 affecting H stability [31]. Our results now show that this domain contains sequences required for the CycG-Hairless interaction as well.

The *in vitro* interactions observed between Hairless and CycG in the yeast system were verified *in vivo* by co-immunoprecipitation experiments. Antibodies directed against Hairless allowed to co-precipitate CycG protein from embryonic protein extracts, and *vice versa*, antibodies directed against CycG co-precipitated Hairless (Fig 1C).

### Loss of CycG affects wing venation phenotypes in Notch pathway mutants

Based on the fact that we had isolated CycG as an interaction partner of the Notch antagonist Hairless, we next addressed potential genetic interactions between mutants affecting the *cycG* gene and several Notch pathway components. To this end we used the null mutant alleles *cycG*^*HR7*^ and *cycG*^*eoC*^ which were generated by homologous recombination [25, 27]. In addition, a third allele *CycG*^*CreD*^ was generated from *cycG*^*HR7*^ that contains only the defective distal gene copy [27]. Mostly the *cycG*^*HR7*^ allele was used for the subsequent experiments if not noted otherwise.

**Fig 2:**
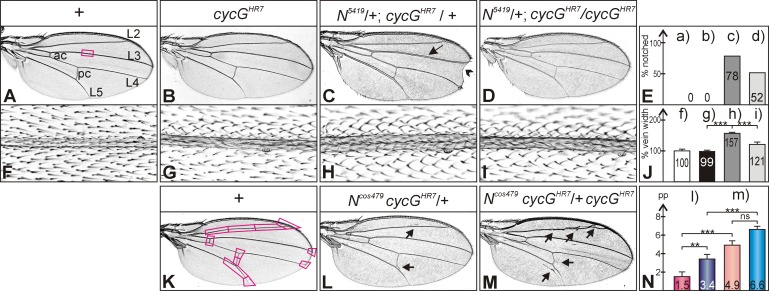
Genetic interactions between *cycG* and *Notch* mutants during wing development. **(A)** Wing of a wild type control (+), characterized by five longitudinal veins (shown are L2 –L5) and an anterior and a posterior crossvein (ac, pc). The square depicts the approximate area enlarged in F)—I). **(B**) Homozygous *cycG*^*HR7*^ mutant wings have a wild type appearance. **(C)** Wings of heterozygous *N*^*5419*^ / +; *cycG*^*HR7*^ / *+* mutant females display notched wing tips with high frequency (arrowhead) and a thickened L3 wing vein (arrow). **(D)** Wings of a female fly *N*^*5419*^ /+; *cycG*^*HR7*^ / *cycG*^*HR7*^ has a more normal appearance. **(E)** Comparison of wing notching: neither wild type (a) nor *cycG*^*HR7*^ / *cycG*^*HR7*^ homozygous flies (b) show notched wings; (c) about 78% of the wings from *N*^*5419*^ / +; *cycG*^*HR7*^ / *+* doubly heterozygotes are notched (n = 646); (d) *N*^*5419*^ /+; *cycG*^*HR7*^ / *cycG*^*HR7*^ females display about 52% notched wings (n = 342). **(F–I)** Thickness of veins was measured on high magnification pictures as shown: F) wild type. G) *cycG*^*HR7*^ / *cycG*^*HR7*^. H) *N*^*5419*^ / +; *cycG*^*HR7*^ / *+*. I) *N*^*5419*^ /+; *cycG*^*HR7*^ / *cycG*^*HR7*^. **(J)** Vein thickness was measured in pixels and is shown relative to wild type (f) which was taken as 100% (n = 20), genotypes (f-i) are as in (F-I). Standard deviation is indicated. Statistical significance was determined by Student’s T-Test (*** p<0.001). **(K)** Wild type wing is shown with 11 positions used to evaluate vein phenotypes: thickened or ectopic veins were given a value of 1, normal veins a value of 0. **(L)** *N*^*cos479*^ /+; *cycG*^*HR7*^ / *+* flies show a gain of function phenotype, typified by ectopic veinlets (arrows point to examples) appearing in about 1–3 positions. **(M)** The absence of CycG enhances the *N*^*cos479*^ vein phenotype, with ectopic and thickened veins appearing in 5–7 positions (genotype, *N*^*cos479*^ /+; *cycG*^*HR7*^ / *cycG*^*HR7*^) (arrows point to examples). **(N)** Numerical evaluation according to (K) of vein defects in (l) *N*^*cos479*^ /+ heterozygous and (m) *N*^*cos479*^ /+; *cycG*^*HR7*^ / *cycG*^*HR7*^ females (reddish colours) and males (bluish colours); (n = 10). The Y-axis represents the total number of phenotypically aberrant positions per wing (pp). Due to a gender bias, the phenotype is stronger in males than in females; female wings are shown in L) and M). Standard deviation is indicated. Statistical significance was determined by Student’s T-Test (ns, not significant [p≥0.5]; ** p<0.01; *** p<0.001).

Mutations in either, *Notch (N)*, *Delta (Dl)* or *Hairless (H)* locus are haplo-insufficient, i.e. a characteristic dominant wing phenotype is observed in heterozygous flies. The following null alleles were used, Df(1)-N5419 [40], *Dl*^*B2*^ [39] and *H*^*P8*^ [37]. In addition, we included the null allele *Su(H)*^*∆47*^ [42] and the Notch duplication *N*^*cos479*^ [41] in our assays. Each heterozygous mutant was analyzed in combination with the homozygous *cycG*^*HR7*^ mutant allele for changes of wing phenotypes. No deviations from wild type control were noted in the combination with *Su(H)*^*∆47*^. However, we observed less wing notches typical for *N*^*5419*^ mutants: whereas about 80% of the wings of the control flies heterozygous for *N*^*5419*^ showed at least a single small notch at the margin, roughly 50% of the wings from *N*^*5419*^ /+; *cycG*^*HR7*^ */ cycG*^*HR7*^ flies did so (Fig 2A–2E). It was brought to our attention that the penetrance of the notched wing phenotype is dependent on the genetic background, which we confirmed for two different wild type backgrounds and for *w*^*1118*^: the numbers varied between ca. 34–50% (see S1 Fig). We hence included two further *cycG* null alleles, *cycG*^*CreD*^ which was derived from *cycG*^*HR7*^ (see Materials and Methods) and *cycG*^*eoC*^ which was generated independently [25]. Compared to their heterozygous siblings, homozygosis of each of the alleles ameliorated the wing notching. It was, however, increased compared to the wild type controls (S1 Fig).

**Fig 3:**
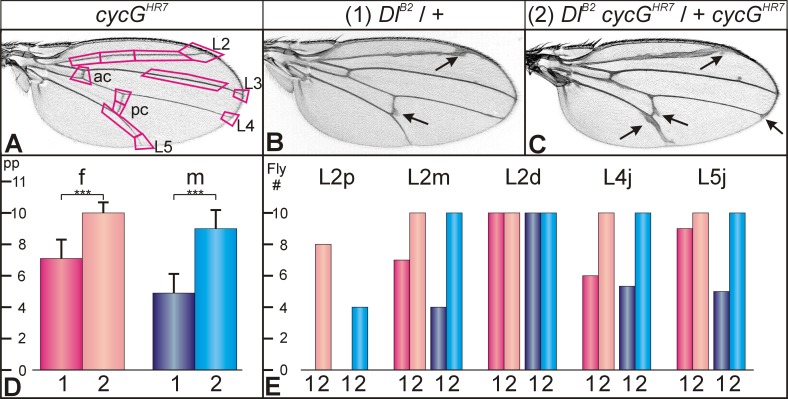
Genetic interactions between *cycG* and *Delta* mutants during wing development. **(A)** Homozygous *cycG*^*HR7*^ mutants have wild type looking wing veins, labelled as in Fig 2. The 12 positions used for phenotypic evaluation are framed. Vein thickening at any of these positions was valued 1, wild type was valued 0. **(B)** Wings of heterozygous *Dl*^*B2*^ / + flies display thickened veins and deltas at the margin; arrows point to some examples. Male wing; the phenotype is generally slightly stronger in females than in males (see D). **(C)** Wings of the combined *Dl*^*B2*^ *cycG*^*HR7*^ / + *cycG*^*HR7*^ flies display an enhanced vein thickening phenotype (female; arrows point to some examples). **(D)** Numerical evaluation according to (A) of wings from *Dl*^*B2*^ / + (1, darker colours) and *Dl*^*B2*^ *cycG*^*HR7*^ / + *cycG*^*HR7*^ (2, lighter colours) flies. Females (f) in reddish, males (m) in bluish colours; (n = 10). The Y-axis represents the values of positions with phenotypic aberration per wing (pp). Statistical significance was determined by Student’s T-Test (*** p<0.001). **(E)** The most sensitive positions of vein thickening in *Dl*^*B2*^ / + mutants that are influenced by loss of CycG are the longitudinal vein L2 and the junction of L4 and L5 at the margin. Given is the number of flies (Fly #, Y-axis) with vein thickening at the respective position; genotypes were *Dl*^*B2*^ / + (1, darker colours) and *Dl*^*B2*^ *cycG*^*HR7*^ / + *cycG*^*HR7*^ (2, lighter colours). L2 thickness was analyzed with regard to the proximo-distal position. The proximal third of L2 (L2p) is only affected in flies lacking *cycG*, i.e. *Dl*^*B2*^ *cycG*^*HR7*^ / + *cycG*^*HR7*^ animals (2), whereas the distal third (L2d) is always affected also in the *Dl*^*B2*^ / + heterozygotes (1). Also shown is a comparison of L4 and L5 junctions (L4j, L5j). Note gender bias of the phenotypes: females in reddish, males in bluish colours. L2p, proximal part of L2; L2m, middle part of L2; L2d, distal part of L2; L4j, L5j, junctions of L4 and L5 with the wing margin; (n = 10).

*N*^*5419*^ heterozygotes display a second typical wing phenotype, namely a thickening of the third and the fifth longitudinal veins, L3 and L5 [40] (Fig 2C and 2H): we measured an increase in the width of L3 to about 160% of the wild type value (Fig 2C, 2H and 2J). Also this vein phenotype was weakened in combination with the homozygous *cycG*^*HR7*^ mutant: the thickness dropped to about 120% of the wild type value (Fig 2F–2J). Whereas the dominant *Notch* wing phenotypes were rescued by loss of CycG, the knotted wing vein phenotype of *N*^*cos479*^ containing an extra copy of the Notch gene was enhanced [41] (Fig 2K–2N). A similar enhancement as a result of *cycG* loss was observed for the dominant wing phenotype of the *Dl*^*B2*^ mutant (Fig 3A–3C). Here, we observed a distinct gender bias in addition (Fig 3D and 3E).

**Fig 4:**
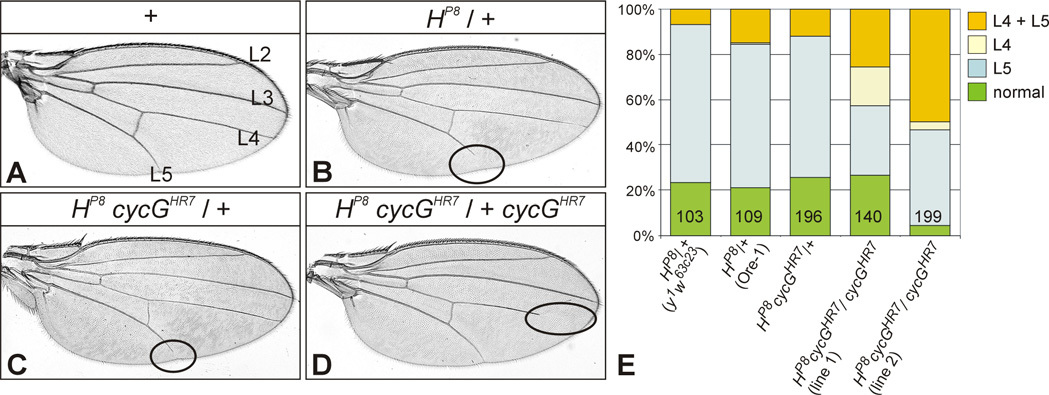
Genetic interactions between *cycG* and *Hairless* mutants during wing development. **(A)** Wing of a wild type fly (Ore-1); longitudinal veins L2-L5 are labelled. **(B)** The wing of a heterozygous *H*^*P8*^/+ mutant female (Ore-1 background) shows the haplo-insufficient *H* phenotype characterized by a gap in the longitudinal L5 vein (encircled). **(C)** In the background of one mutant *cycG*^*HR7*^ copy, the *H* wing phenotype is similar to the Ore-1 background (see also E). **(D)** Wing of a female fly of the genotype *H*^*P8*^ *cycG*^*HR7*^ / *+ cycG*^*HR7*^. Note complete L5 vein but gap in L4 (encircled). In general the venation phenotype *H*^*P8*^ of appears enhanced in the homozygous *cycG*^*HR7*^ background; the fraction of flies bearing a gap in L4 is strongly increased (see also E). **(E)** Graph showing the percentage of wings with vein gaps in *H*^*P8*^ female flies with different genetic backgrounds. Heterozygous *H*^*P8*^ females show normal venation in about 20% of the wings; in the others mostly L5 is affected (63–70%), L4 in addition much less frequently (7–15%). Rarely, L4 is solely affected (1 wing of 109 in *H*^*P8*^ /+). In the homozygous *cycG*^*HR7*^ background, the fraction of wings where both, L4 plus L5 are affected is increased (26–50%). Notably, the peculiar phenotype of gaps in L4 only is seen in up to 36% of the flies. Two different recombinant stocks (lines 1, 2) were analyzed, which showed a similar effect albeit with some variations. Female flies were analysed. Number of wings analyzed is given in each column.

Flies heterozygous for the *Hairless* null allele *H*^*P8*^ display a shortening of the L5 vein in about 60–70% of the wings (Fig 4A and 4B) [37]. Dependent on the genetic background, L4 might be affected in addition with a lower penetrance (7–15%) (Fig 4E). These numbers are very similar in heterozygous *H*^*P8*^ *cycG*^*HR7*^ /+ flies (Fig 4C and 4E). In the absence of CycG, i.e. in the *H*^*P8*^ *cycG*^*HR7*^ */ + cycG*^*HR7*^females, the vein phenotype was enhanced, since the number of wings where both L4 and L5 are affected was clearly increased (26–50%). Notably, the number of wings only displaying a gap in the L4 vein, which was very rare in *Hairless* heterozygous females (0.9%), was seen more often in the absence of *cycG* (4–17%) (Fig 4D and 4E). Two different recombinant lines were assayed to account for the influence of the genetic background. Taken together, mutants of *cycG* and Notch pathway members display intimate genetic interactions with regard to wing development.

### Genetic interactions of *cycG*^*HR7*^ with the EGFR network during wing vein development

**Fig 5:**
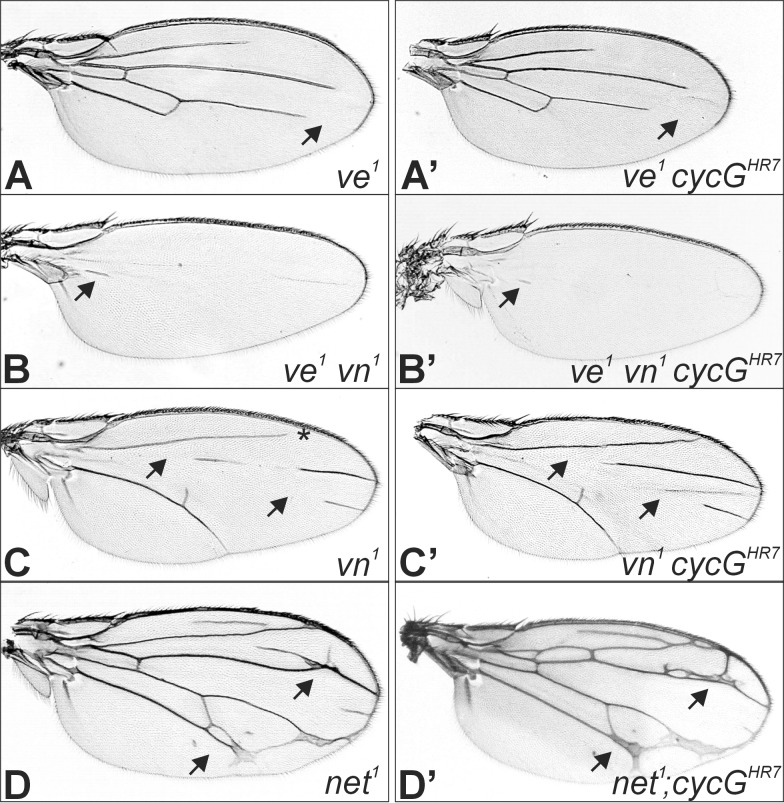
Genetic interactions between mutants in *cycG* and EGFR pathway members during wing development. **(A)** The *ve*^*1*^ mutant phenotype is characterized by distal gaps in the longitudinal veins (arrow points to L4). **(A’)** In the doubly mutant *ve*^*1*^ *cycG*^*HR7*^, the gap in L4 appears slightly larger (arrow). **(B)** Wings from doubly mutant *ve*^*1*^ *vn*^*1*^ flies lack all veins but small remains (arrow). **(B’)** This phenotype is not altered in the *cycG*^*HR7*^mutant. **(C)** The *vn*^*1*^ mutant wing has gaps in the longitudinal veins L3 and L4 (arrows), and frequently L2 does not reach the margin (asterisk) (Genotype is: *ve*^*1*^ *vn*^*1*^/*vn*^*1*^ *cycG*^*HR7*^*)*. **(C’)** Wings of doubly mutant *vn*^*1*^ *cycG*^*HR7*^ flies display smaller gaps (arrow). **(D)** The *net*^*1*^ mutant wing is characterized by a network of veins (arrow points to examples) (Genotype is: *net*^*1*^*/net*^*1*^; *cycG*^*HR7*^*/TM6B)*. **(D’**) More extra veins are observed in the doubly mutant *net*^*1*^*; cycG*^*HR7*^ (arrow).

During wing vein development the EGFR- and Notch pathways are closely intertwined: EGFR signalling is required early on to induce pro-vein potential which is subsequently limited by Notch activity to the veins proper (reviewed in [5, 49]). Based on our earlier observations of a genetic interaction between CycG and the EGFR signalling pathway during oogenesis [28], we asked whether *cycG* mutants might influence EGFR activity during wing vein formation. To address this question, mutant alleles of three genes, *net (net*^*1*^*)*, *veinlet (ve*^*1*^, also named *rhomboid*, *rho*^*1*^) and *vein (vn*^*1*^*)* were analyzed. These genes are known to act upstream of EGFR during vein formation [5, 49]: Whereas Vn acts as EGFR ligand within the proximal wing, Ve is required for EGFR activity within the distal wing [49]. Accordingly, single mutants lack the veins in the respective area, whereas the double mutants lack veins altogether [43] (see Fig 5A–5C). In contrast, Net is required to restrict Ve activity (reviewed in [49]) and the *net*^*1*^ mutant allele shows a mesh of ectopic veins [50] (see Fig 5D). Mutants of either *ve*^*1*^, *vn*^*1*^, *net*^*1*^ or the double mutant *ve*^*1*^ *vn*^*1*^ were combined or recombined with the *cycG*^*HR7*^ allele to investigate for phenotypic alterations in the doubly homozygous mutants. The observed effects were small and inconclusive. Whereas the phenotype of the double mutant *ve*^*1*^ *vn*^*1*^ was unchanged by a loss of CycG, a slight enhancement was observed in the *ve*^*1*^ *cycG*^*HR7*^ double mutant, which was however, restricted to the L4 vein (Fig 5A and 5B’; S2 Fig). In contrast, loss of CycG mildly ameliorated the phenotype of the *vn*^*1*^ mutant (Fig 5C and 5C’; S2 Fig). In the combination with *cycG*^*HR7*^ the *net*^*1*^ phenotype was slightly enhanced (Fig 5D and 5D’; S2 Fig). However, the *net*^*1*^ phenotype appears highly susceptible to genetic background, as it was likewise enhanced by the unrelated mutations *speck (sp*^*1*^*)* and *roughoid (ru*^*1*^*)* (S2 Fig). Hence, an influence of *cycG*^*HR7*^ on *net*^*1*^ could not be conclusively demonstrated.

Since Ve and Net are known antagonists [43], the enhancement of either phenotype by loss of CycG was unexpected. Moreover, the opposite effect of CycG on Ve and Vn which are both involved in EGFR activation—Ve during release of the ligand Spitz, and Vn as a secreted ligand itself [5, 49]—cannot be easily reconciled. Since the phenotypic alterations were subtle, they may be indirect and may well result from a modulation of the Notch signalling pathway by CycG.

### Influence of *cycG* mutants on Notch mediated processes during oogenesis

**Fig 6:**
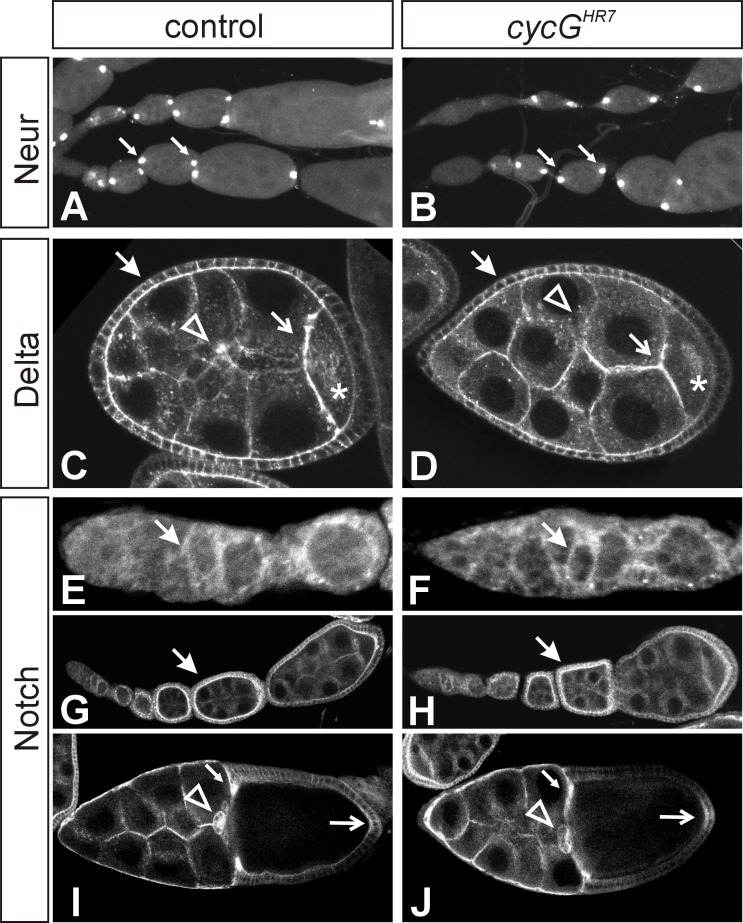
Notch dependent processes during early stages of oogenesis appear unaffected in *cycG* mutant ovaries. Wild type **(A, C, G, I)** and *cycG*^*HR7*^ homozygous mutant **(B, D, H, J)** egg chambers and germaria **(E, F)** are shown; expression of each marker appears the same in both genotypes. **(A, B)** Expression of *neur*^*A101*^*-lacZ* reporter line is restricted to the two polar cells (arrows) in each egg chamber. Two ovarioles are shown for each genotype. **(C, D)** Delta protein is most highly expressed in egg chambers between stage 5 and 7. Delta protein preferentially accumulates along the membranes: between follicle cells (closed arrow), between follicle cells and nurse cells, and between nurse cells and the oocyte (open arrow). Delta protein is also seen in the cytoplasm of the oocyte (asterisk) and the nurse cells, where it is enriched in dots (arrowhead). Posterior follicle cells overlying the oocyte display a marked downregulation of Delta expression. **(E-J)** Ovarioles were stained with antibodies directed against the intracellular domain of Notch. (**E, F)** Notch protein appears slightly enriched in the arising somatic follicle cells within the germarium (arrow). (**G, H)** Note accumulation of Notch protein along the membranes of follicle cells, which is highest in stage 5 to 7 egg chambers (arrow). **(I, J**) In a stage 10 egg chamber, Notch protein is most strongly detected in the migrating border cells (arrowhead), the centripetal cells (small arrow) and in the posterior follicle cells (open arrow).

The primary phenotype of *cycG* mutants is female sterility and a ventralization of the eggshell [27, 28]. However, these phenotypes were unchanged in the combination of *cycG*^*HR7*^ homozygotes with either *Notch*, *Delta* or *Hairless* heterozygous mutants, suggesting that these phenotypes may be unrelated to Notch signalling. Notch signalling is involved in several steps during oogenesis. Notch activity is already required during the formation of the stem cell niche [51, 52]. Later on, the formation of two polar cells, located at the anterior and the posterior pole of each follicle, and the stalk cells separating the egg chambers, depends on Notch activity [8]. In *cycG* mutant ovaries polar cells developed normally as visualized with a *neur*^*A101*^-lacZ reporter line (Fig 6A and 6B); the stalk cells appeared normal as well. During stages 5 to 7 of oogenesis, a strong Delta signal is sent by the oocyte to pattern the overlying follicle cells [8]. Accordingly, Delta protein is found enriched along the membranes between the nurse cells and the oocyte as well as between the nurse cells and the follicle cells [53] (see Fig 6C). Overall, the Delta protein expression pattern appeared normal in the *cycG* mutant egg chambers (Fig 6D). Likewise, Notch protein is visible in germaria and becomes enriched in follicle cells of stages 5 to 7 egg chambers (see Fig 6E and 6G). Later on, Notch protein is found at highest concentrations in the migrating centripetal and border follicle cells, and also within the posterior follicle cells [54] (see Fig 6I). Again, no differences were seen between the *cycG* mutant and the control (Fig 6E–6J). These results indicate that the female sterility affiliated with a loss of CycG is not caused by an impaired Notch signalling activity.

### Notch protein expression during dorsal appendage formation is affected in *cycG* mutant egg chambers

**Fig 7:**
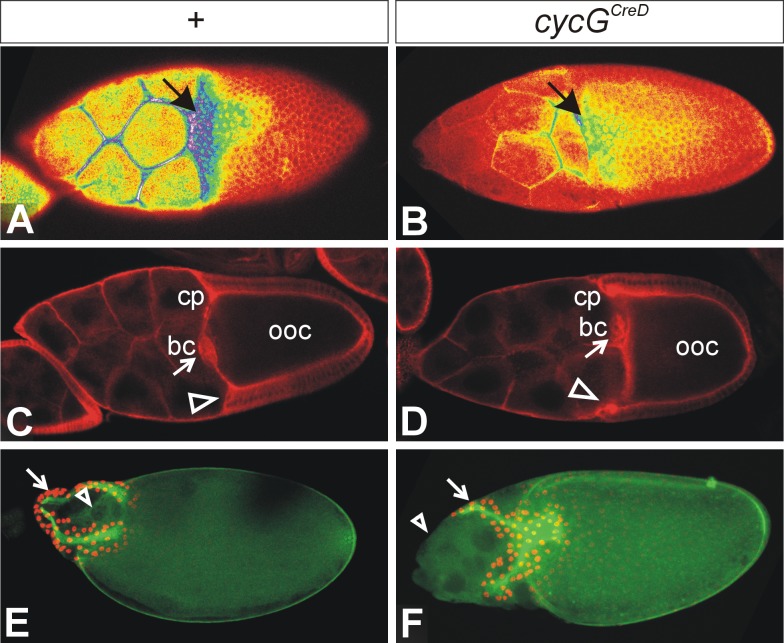
Notch expression and nurse cell positioning are affected in *cycG* mutant ovaries. **(A, B)** Superficial view onto a stage 10 egg chamber stained for Notch protein. Shown is a false colour picture with colours representing strength of signal from red (low) to yellow (intermediate) to blue (strong). Pictures were taken with identical settings from ovaries stained in parallel experiments with identical conditions. **(A)** In the wild type, Notch is expressed in a graded fashion and is mostly enriched in the prospective floor cells of the developing dorsal appendages (arrow). **(B)** In the *CycG*^*CreD*^ homozygous mutant, Notch expression is markedly reduced in the entire follicle (arrow). **(C, D)** A sagittal view shows the enrichment of Notch protein in the centripetal cells (cp; arrowhead) and the border cells (bc; arrow) at the anterior border of the oocyte (ooc). The anterior border is slightly convex or straight in the wild type **(C). (D)** In the *cycG*^*CreD*^ homozygote, the nurse cells push into the oocyte, i.e. the centripetal cells (cp; arrowhead) are located anterior relative to the border cells (bc; arrow). **(E, F**) A superficial view onto stage 13–14 egg chambers is shown. The developing dorsal appendages (arrow) are marked with anti-Broad antibodies (nuclear, red); the green staining of the eggshell is due to auto-fluorescence. In the wild type **(E),** the nurse cells have been eliminated by apoptosis (arrowhead). **(F)** Egg chambers of homozygous *cycG*^*CreD*^ females frequently show a ‘dumpless’ phenotype, because extant nurse cells are found outside of the oocyte beyond stage 14 (arrowhead).

The dorsal appendages can be traced back to two cell populations, the dorsal roof and the ventral floor cells [55]. The dorsal roof cells are specified by the expression of the transcription factor Broad, which is regulated in the stage 10 egg chamber through the EGFR- and DPP-signalling pathways in complex feedforward and feedback loops [56]. We have observed before that Broad expression is frequently fused to a single domain in *cycG* mutant egg chambers anticipating the fused dorsal appendages seen in mutant eggs [28]. The ventral floor cells are specified by the expression of *rhomboid*, whereas the boundary between ventral floor and dorsal roof cells is established by Notch signalling activity [57]. Accordingly, Notch protein is enriched in the dorsal most follicle cells separating the two Broad expression domains [57] (see Fig 7A). We noted that Notch protein expression was strongly reduced in the *cycG* mutant egg chamber (Fig 7B). However, at this stage, appendages are already determined, and the alterations of Notch protein distribution cannot explain a fusion of the dorsal appendages. It seems also unlikely that the downregulation of Notch protein in these cells is a consequence of the appendage fusion. This suggests an additional, later role for *cycG* in dorsal floor cell fate acquisition. However, the primary defect is within the patterning of the oocyte mediated by a reduced *grk* signal [27, 28] and hence, at the level of EGFR signalling activity. Therefore it seems more likely that the downregulation of Notch protein expression is a result of the downregulation of EGFR activity within the dorsal follicle cells.

Apart from the reduced Notch protein accumulation in *cycG* mutant stage 10 egg chambers, we also noted a change in oocyte morphology. The stages 9 and 10 are characterized by specific movements of the follicle cells and by a strong growth of the oocyte (reviewed in [58, 59]). By stage 10 columnar follicle cells cover only the oocyte, whereas stretched follicle cells span the nurse cells (exemplified in the control staining of Fig 7C). In the late stage 10 egg chamber, once the border cells have reached the oocyte, the centripetal follicle cells start their inward movement to eventually cover the oocyte anteriorly (reviewed in [58, 59]). The border between oocyte and anterior follicle cells is convex or straight (Fig 7C), which is not the case in the *cycG*^*CreD*^ mutant egg chambers (Fig 7D). Here, the columnar follicle cells appear to stop early, partly covering the posterior most nurse cells. In contrast, the border cells migrated as expected until touching the oocyte (Fig 7D). Hence, border cells lie posterior with respect to the ingressed centripetal cells. As shown before [60], formation of squamous stretched follicle cells depends on the disassembly of the adherens junctions, a prerequisite for the posterior displacement of the main body follicle cells. This requires Notch signalling, as the latter is delayed in its absence [60, 61]. The observed defects in follicle cell patterning in the *cycG*^*CreD*^ mutant egg chambers are compatible with the proposed positive role of CycG for Notch activity also during this process.

During stages 11 and 12, the nurse cells dump their content into the oocyte to eventually die (reviewed in [58, 59]). In about 15% of *cycG*^*CreD*^ mutant egg chambers (31 out of 201) this process was incomplete, and nurse cells remained attached to the maturing egg (Fig 7E and 7F).

## Discussion

### CycG modulates Notch activity during wing development

In this work, we have identified a molecular and genetic link between CycG and the Notch signalling pathway during wing development and late stages of oogenesis. We have shown that CycG protein binds to Hairless protein *in vitro* and *in vivo*. Hairless is the major antagonist of Notch signalling in the fly, repressing Notch target gene transcription in the absence of Notch receptor activation (reviewed in [30]). Based on our results, we propose that CycG may promote Hairless activity by direct contact, thereby restricting Notch signalling activity during wing vein refinement.

Vein width in the *Drosophila* wing is regulated by the Notch pathway, which determines the border between vein and intervein fate in the presumptive pro-vein territory [4, 5, 49]. Initially vein fate is promoted by EGFR signalling activity resulting in the expression of Delta within the pro-vein territory. Delta then represses vein fate non-autonomously in lateral pro-vein cells by activating the Notch receptor, causing the repression of EGFR activity. Hairless, by antagonizing Notch signalling, protects presumptive vein cells from aberrant Notch signals. Delta expression, however, is negatively regulated in a complex circuitry by Notch signals [62]. The genetic interactions of *cycG* and *H* mutants are in agreement with the idea that CycG acts as a positive mediator of Hairless, since the vein phenotype of the *Hairless* heterozygotes is aggravated in the absence of CycG. Maybe binding of CycG protein enforces the function of the Hairless repressor complex, thereby increasing the threshold for the transcriptional activation of Notch target genes. Accordingly, the vein thickening in *N* heterozygotes is ameliorated in the absence of CycG, since the now reduced signalling activity of Notch is more likely to overcome the repressor barrier. However, the genetic data also support a role of CycG as a positive modulator of Delta, since in its absence the *Delta* knotted vein phenotype is enhanced. This apparent contradiction may be resolved taking into account the complex regulatory network, where Hairless acts indirectly as an activator of Delta gene expression [62]. If CycG promotes Hairless activity, its absence is expected to enhance the effects of a Delta loss, which we indeed observed. Extra gene doses of *Notch* (e.g. *N*^*cos479*^), however, cause phenotypes very similar to a loss of Delta [41, 63, 64]. Moreover, these phenotypes are enhanced in the absence of CycG. Interestingly, these apparent dominant negative phenotypes can also be observed by extra copies of just the extracellular domain of Notch [63, 64], as for example in the *N*^*Co*^ mutation [65], indicating that the extracellular domain of the Notch receptor is sufficient to effectively titrate the Delta ligand. Accordingly, loss of one gene copy of *Notch* ameliorates the heterozygous *Delta* wing phenotypes and vice versa, instead of aggravating it [66, 67]. It has been proposed that Delta ligand and Notch receptor present within the same cell titrate each other, a process termed *cis*-inhibition, which has been also observed during photoreceptor specification and wing margin formation in *Drosophila* (reviewed in [68]). Cis-inhibition may enforce the directionality of Delta-Notch signalling in the process of vein width restriction [68]. The improvement of the *Notch* mutant wing phenotype, and likewise the enhancement of vein knotting in *N*^*cos479*^ wings by the absence of CycG would hence be expected, if CycG acts indirectly as a positive mediator of Delta: its absence would result in a quenching of Delta activity, formally similar to a dose reduction of the *Delta* gene and expected to rescue loss of Notch by the titration effect.

We have recently shown that the *cycG* mutants are impaired in growth and lipid metabolism. In this context, CycG acts as positive regulator of InR/TORC1 signalling at the level of Akt1 kinase presumably via a regulation of PP2A-Akt1 binding [25]. The Thr/Ser Phosphatase PP2A is a known negative regulator of Akt1 kinase [69, 70]. We have observed that mutants of the PP2A B’ subunit *widerborst* rescue the growth defects of the *cycG* mutants [15]. Interestingly, Widerborst inhibits Notch signalling during the early retinal development [71]. One may speculate that a loss of CycG results in an increased Widerborst activity also during wing development, thereby effecting a reduced Notch signalling activity which is reflected by the genetic interactions we have observed.

### CycG involvement in later stages of egg formation

In the germ line, CycG is required for timely meiotic recombination repair. Accordingly, *cycG* mutants display phenotypes typical of genes defective in DNA double strand repair. CycG is found in the BRCA2/ 9-1-1 protein complex involved in the sensing of double strand breaks and the loading of repair enzymes onto damaged DNA [27, 72]. In addition to its early role in the germ line, CycG appears also to be required for normal egg chamber morphology. In addition to dorsal appendage fusions in *cycG* mutant eggs resultant from an impaired EGFR signalling activity [27, 28], we now find a reduced accumulation of Notch protein and also a slower migration of the columnar follicle cells, which requires Notch signalling activity as well [60, 61]. Apparently, CycG is not only important for full Notch activity during the development of the wing but also of the egg chamber at late stages of oogenesis. We cannot exclude that the latter defects are a consequence of the reduced EGFR activity resulting from the early phase of CycG requirement. In the light of the specific role of CycG for Notch activity during wing development, however, it is conceivable that CycG plays a likewise specific role for Notch activation during the morphogenesis of somatic follicle cells. Further experiments are required to distinguish between these possibilities. Altogether, these data demonstrate intimate genetic interactions amongst Notch pathway members and CycG during wing development.

## Supporting Information

S1 Fig Influence of genetic background on wing notching in heterozygous *N*^*5419*^ /+ females. (PDF)

S2 Fig Genetic interactions of *cycG*^*HR7*^ and *ve*^*1*^, *vn*^*1*^ *ve*^*1*^, and *net*^*1*^ mutants. (PDF)
