# Supplementary material for: Drosophila Cyclin G Is a Regulator of the Notch Signalling Pathway during Wing Development
Source: PLoS One. 2016 Mar 10;11(3):e0151477. doi: 10.1371/journal.pone.0151477 (PMC4786218; doi:10.1371/journal.pone.0151477)
Supplement: S1 Fig — (PDF) [file pone.0151477.s001.pdf]

Supporting Figure S1

Influence of genetic background on wing notching in heterozygous *N*^{5419} /+ females

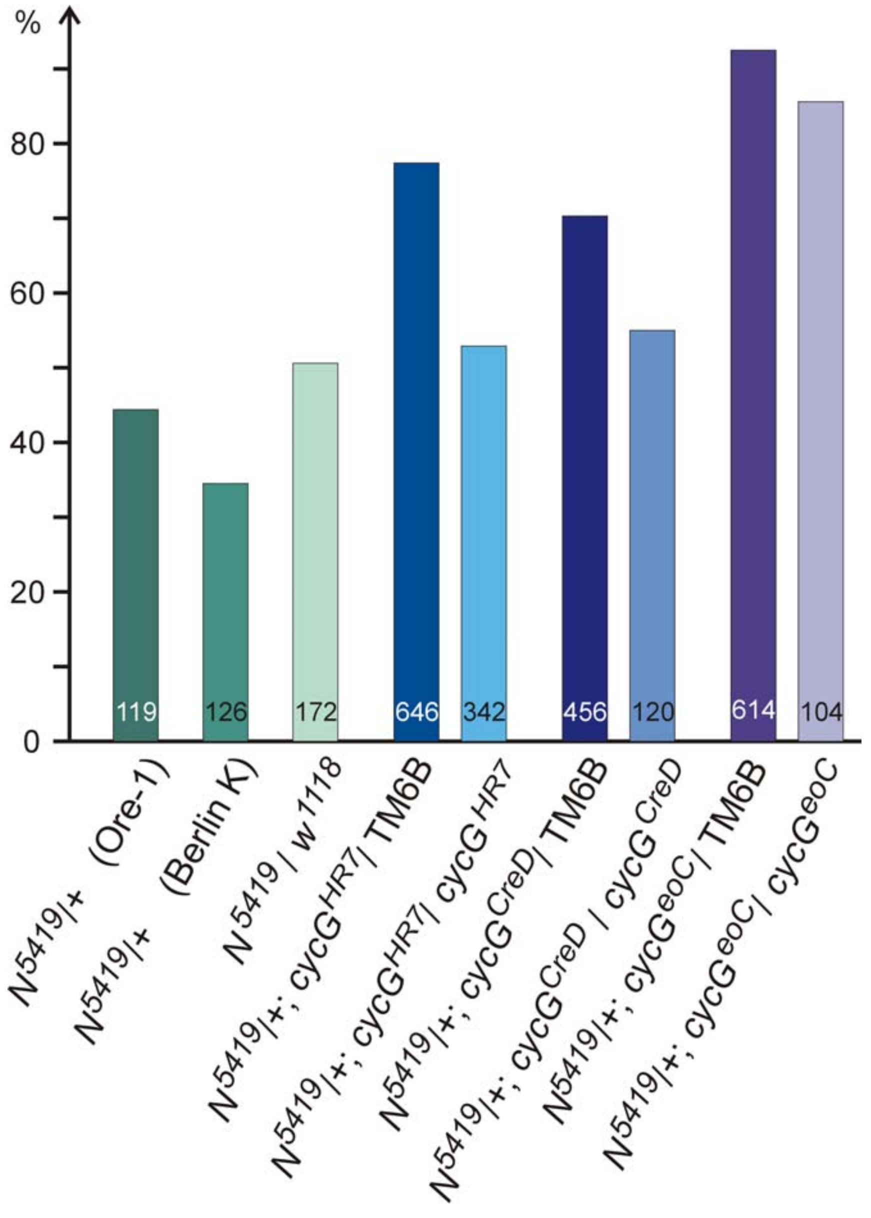

The percentage of heterozygous *N*^{5419} females with notched wings was recorded in three different control backgrounds, Oregon-1 (Ore1), Berlin K and *w*^{1118} (greenish coloration): it varies between ca. 35-51%.

The influence of CycG loss on the *Notch* wing phenotype was addressed using three different *cycG* null alleles, *cycG*^{HR7}, *cycG*^{CreD} and *cycG*^{eoEC}. Compared with the heterozygous siblings balanced over TM6B, the phenotype was always ameliorated in the *cycG* homozygotes. It was, however, markedly stronger compared to the controls. Total number of analyzed wings is given in each column.
