# Supplementary material for: Drosophila Cyclin G Is a Regulator of the Notch Signalling Pathway during Wing Development
Source: PLoS One. 2016 Mar 10;11(3):e0151477. doi: 10.1371/journal.pone.0151477 (PMC4786218; doi:10.1371/journal.pone.0151477)
Supplement: S2 Fig — (PDF) [file pone.0151477.s002.pdf]

Supporting Figure S2

Genetic interactions of *cycG*^{HR7} and *ve*^{1}, *vn*^{1} *ve*^{1}, and *net*^{1} mutants

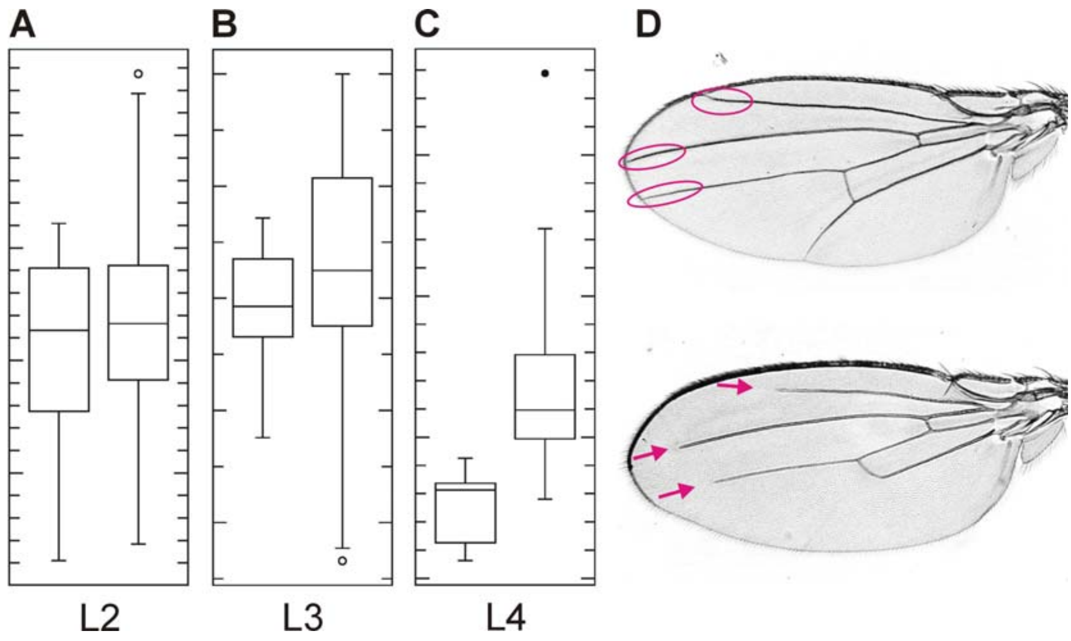

A-D) CycG dependent changes of the *ve*^{1} venation phenotype. Gaps in longitudinal veins L2 (A), L3 (B) and L4 (C) were measured relative to the overall length of the wing. The homozygous *ve*^{1} phenotype (left bars, n=21) was not significantly altered in the absence of *cycG* (*ve*^{1} *cycG*^{HR7}, right bars n=29) except for the gap in L4 which was significantly larger in the double mutant ($p<0.0001$ by Student's T-test). D) Position of gaps highlighted in a control and in a *ve*^{1} mutant wing.

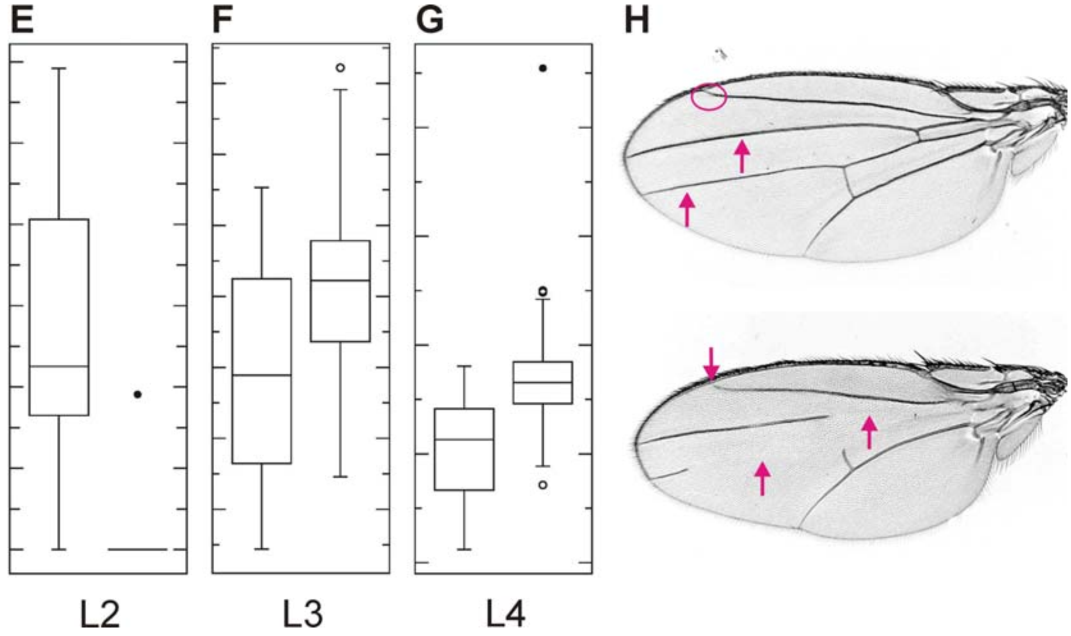

E-H) CycG rescues the vn^{1} venation phenotype. Gaps in the longitudinal vein L2 (E), and remains of longitudinal veins L3 (F) and L4 (G) were measured relative to the overall length of the wing, as demonstrated in a control wing (H, upper panel). The homozygous vn^{1} phenotype (left bars, n=32) was ameliorated in the absence of *cycG* (vn^{1} $cycG^{HR7}$, right bars n=31) ($p<0.0001$ by Student’s T-test); an example is shown in H) (lower panel).

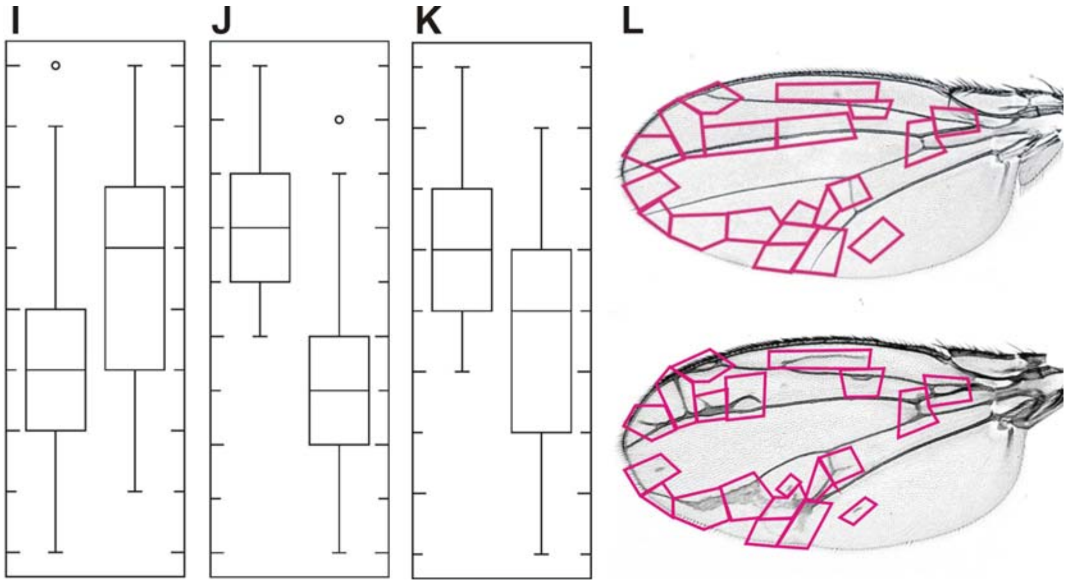

I-L) The net^{1} venation phenotype is highly succeptible to genotype. Ectopic venation was determined in 20 positions as shown in L (upper panel is control, lower panel is an example of a homozygous net^{1} mutant wing).

I) A comparison of net^{1} ; $cycG^{HR7}$ / TM6B (left bar) with the doubly mutant siblings net^{1} ; $cycG^{HR7}$ (right bar) reveals a significant enhancement of the *net* phenotype upon loss of *cycG*.

J) An even stronger, highly significant difference is observed between two net^{1} strains with a different genotype. Left bar represents net^{1} sp^{2} ; ru^{1} and right bar net^{1} ; $cycG^{HR7}$ / TM6B.

K) Accordingly, comparison of net^{1} sp^{2} ; ru^{1} with net^{1} ; $cycG^{HR7}$ shows a mild rescue by loss of *cycG*.
